# Legionella-induced suppurative cervical lymphadenitis in a child diagnosed by metagenomic next-generation sequencing: a case report

**DOI:** 10.3389/fped.2025.1655298

**Published:** 2025-11-07

**Authors:** Bowen Li, Yaru Liao, Jian Li

**Affiliations:** 1 Department of Pediatric Surgery, Jinan Children’s Hospital, Children’s Hospital Affiliated to Shandong University, Jinan, China; 2 Department of Infectious Diseases, Jinan Children’s Hospital, Children’s Hospital Affiliated to Shandong University, Jinan, China

**Keywords:** *Legionella pneumophila*, suppurative lymphadenitis, cervical lymphadenopathy, metagenomic next-generation sequencing, extrapulmonary infection

## Abstract

*Legionella pneumophila*, primarily associated with respiratory infections, rarely causes extrapulmonary disease. Conventional diagnostic methods for *Legionella* are often limited. Here we report a case of suppurative cervical lymphadenitis caused by *L. pneumophila*. And we highlight the critical role of mNGS in enabling rapid and accurate pathogen identification, guiding effective targeted therapy for rare and challenging infections.

## Introduction

The term “*Legionella*” originated from a major public health event in 1976 during an American Legion convention in Philadelphia, where a severe pneumonia outbreak occurred. Through thorough postmortem investigations, medical researchers identified a novel pathogen isolated from affected lung tissues and subsequently designated it *Legionella*. Belonging to the aerobic Gram-negative bacilli family. Among these, *Legionella pneumophila* remains the most the most frequently involved pathogen in human infections (1).

*Legionella* thrives in warm, humid environments and proliferates in both natural and engineered water systems. Transmission occurs primarily through inhalation of contaminated aerosols or, less commonly, ingestion of contaminated water. Exposure risks are heightened by contact with contaminated air-conditioning systems, cooling towers, or potable water, as well as activities such as hot spring bathing, gardening, plumbing work, or recent travel to high-risk areas (2).

*Legionella* infection in humans manifests in three primary forms: Legionella pneumonia, Pontiac fever, and soft tissue infections. The most severe presentation, Legionella pneumonia, is characterized by acute atypical pneumonia marked by acute fibrino-purulent inflammation with rapid progression and high mortality rates. Patients typically develop prodromal symptoms such as headache, myalgia, fatigue, and anorexia, followed by hallmark features including fever, productive cough, and pleuritic chest pain. Beyond pulmonary involvement, the infection often causes multi-system complications affecting the gastrointestinal tract, nervous system, renal system, cardiovascular system, and musculoskeletal tissues (3). Additionally, cutaneous manifestations such as diffuse maculopapular rashes may occur in a minority of cases.

Conventional culture-based methods often fail to detect *Legionella* due to its fastidious growth requirements, posing diagnostic challenges. Metagenomic next-generation sequencing (mNGS), a high-throughput sequencing technology capable of identifying rare and novel pathogens, has emerged as a powerful tool for rapid and accurate pathogen detection (4).

While *Legionella* infections predominantly manifest as respiratory illnesses, extrapulmonary infections remain rare. Here, we present a case of L. pneumophila causing suppurative cervical lymphadenitis in a child diagnosed by mNGS.

## Case presentation

A 5-year-and-4-month-old boy was admitted on August 8, 2024, presenting with recurrent fever and neck swelling and pain persisting for over 10 days. The patient initially developed sudden-onset fever (peak temperature 38.1°C) without chills or seizures. Symptoms temporarily resolved with symptomatic treatment but recurred hours later. Concurrent neck pain and swelling were noted without skin erythema or fluctuance. Negative findings included absence of cough, vomiting, diarrhea, headache, rash, night sweats, or joint swelling. Prior treatment at a local hospital included sequential antibiotic therapy (cefuroxime for 8 days followed by cefoperazone-sulbactam for 1 day) combined with antiviral therapy (potassium sodium dehydroandrograpolide succinate for 9 days) and short-term dexamethasone (3 days). Fever resolved temporarily after 2 days of treatment but recurred once on August 3 before complete resolution. Neck tenderness persisted despite clinical improvement. The child had a travel history to other places within the nearly two weeks before the onset of the disease, but there was no special family history. Physical examination revealed bilateral cervical masses: left-sided 2.5 × 3.0 cm tender non-fluctuant mass, and right-sided 4.0 × 3.0 cm non-tender mass. Bilateral tonsils showed grade I enlargement. Laboratory findings demonstrated leukocytosis (WBC: 13.31 × 10⁹/L) with neutrophilia (70.4%), elevated inflammatory markers (CRP: 40.39 mg/L, ESR: 101 mm/h), and EBV serology patterns suggesting past infection (VCA-IgG >750 U/ml, EBNA-IgG >600 U/ml) without acute-phase antibodies (VCA-IgM and EA-IgG negative). Tuberculosis screening (T-SPOT.TB) was negative. Cervical ultrasound (July 29, 2024) confirmed bilateral lymphadenopathy, more prominent on the right side.

**Figure 1:**
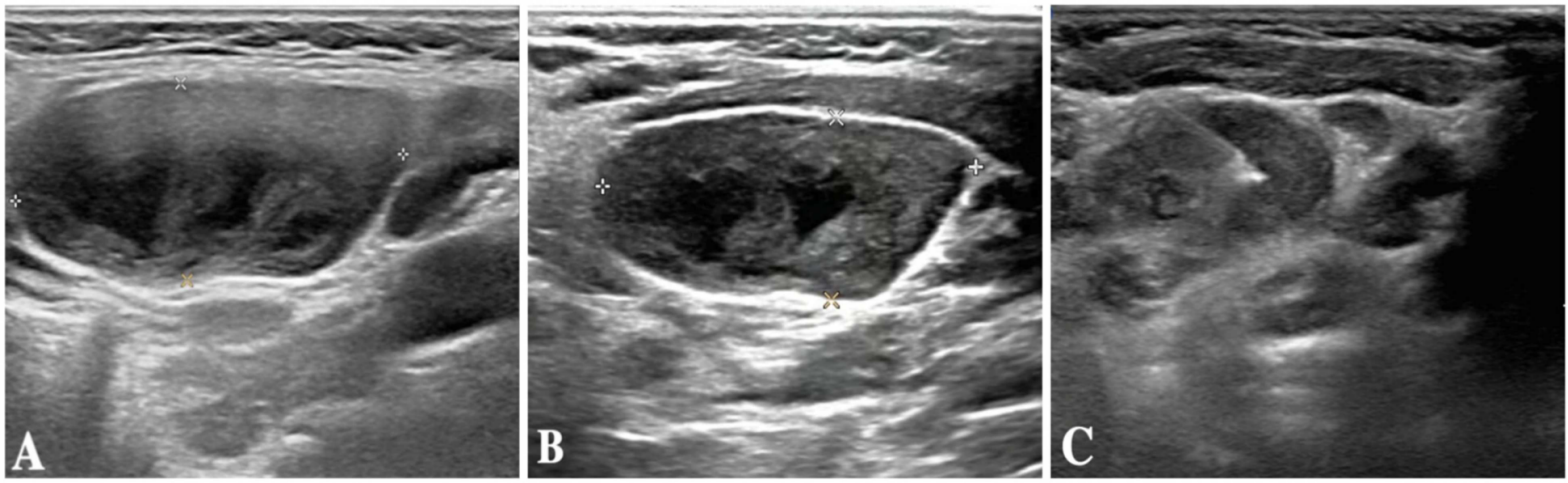
**(A)** Neck ultrasound results of the patient on August 7, 2024. **(B)** Neck ultrasound results of the patient on August 12, 2024. **(C)** Neck ultrasound results of the patient on August 15, 20.

**Figure 2:**
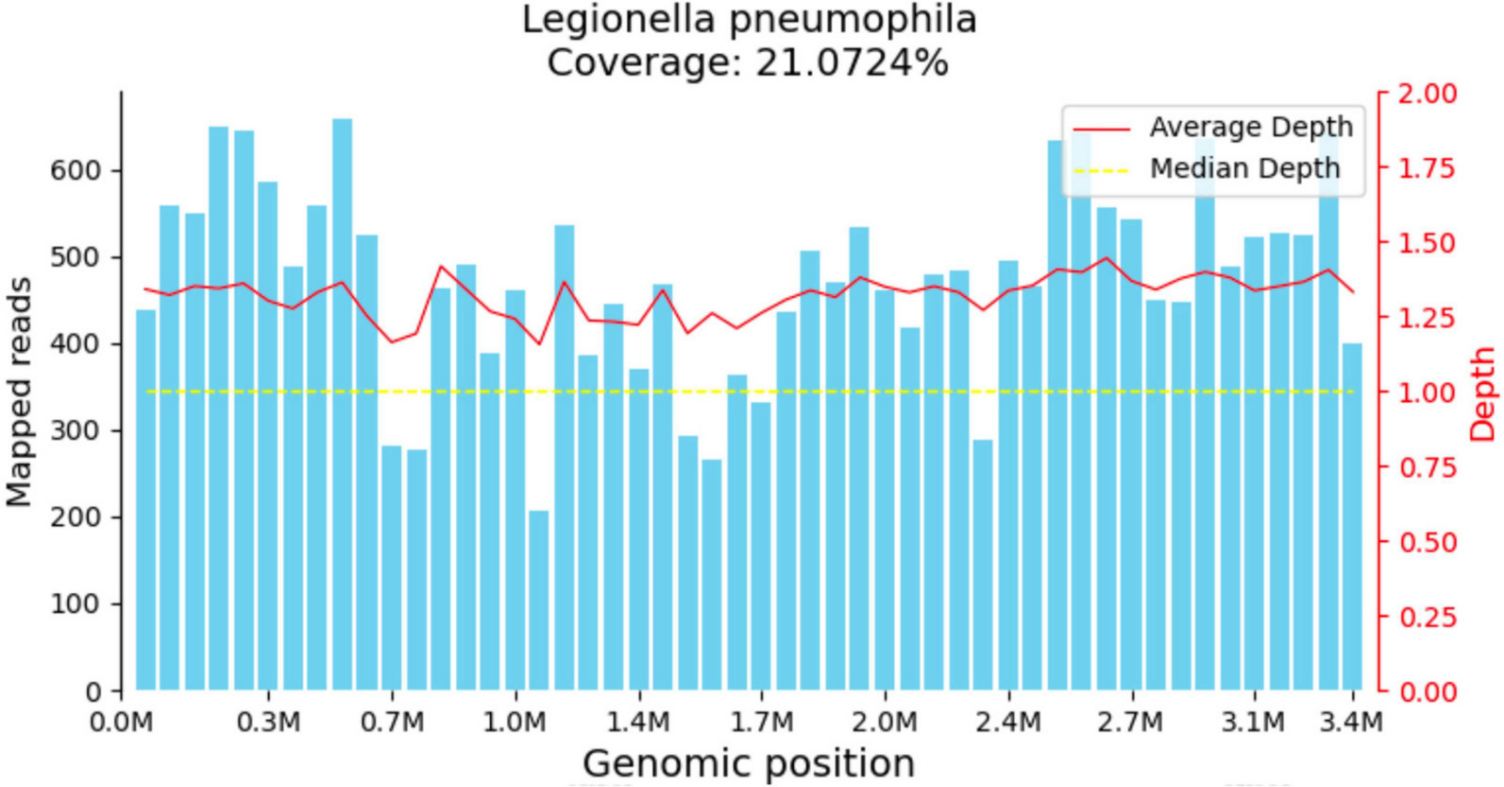
Legionella pneumophila coverage from mNGS of the cervical lymph node pus.

**Figure 3:**
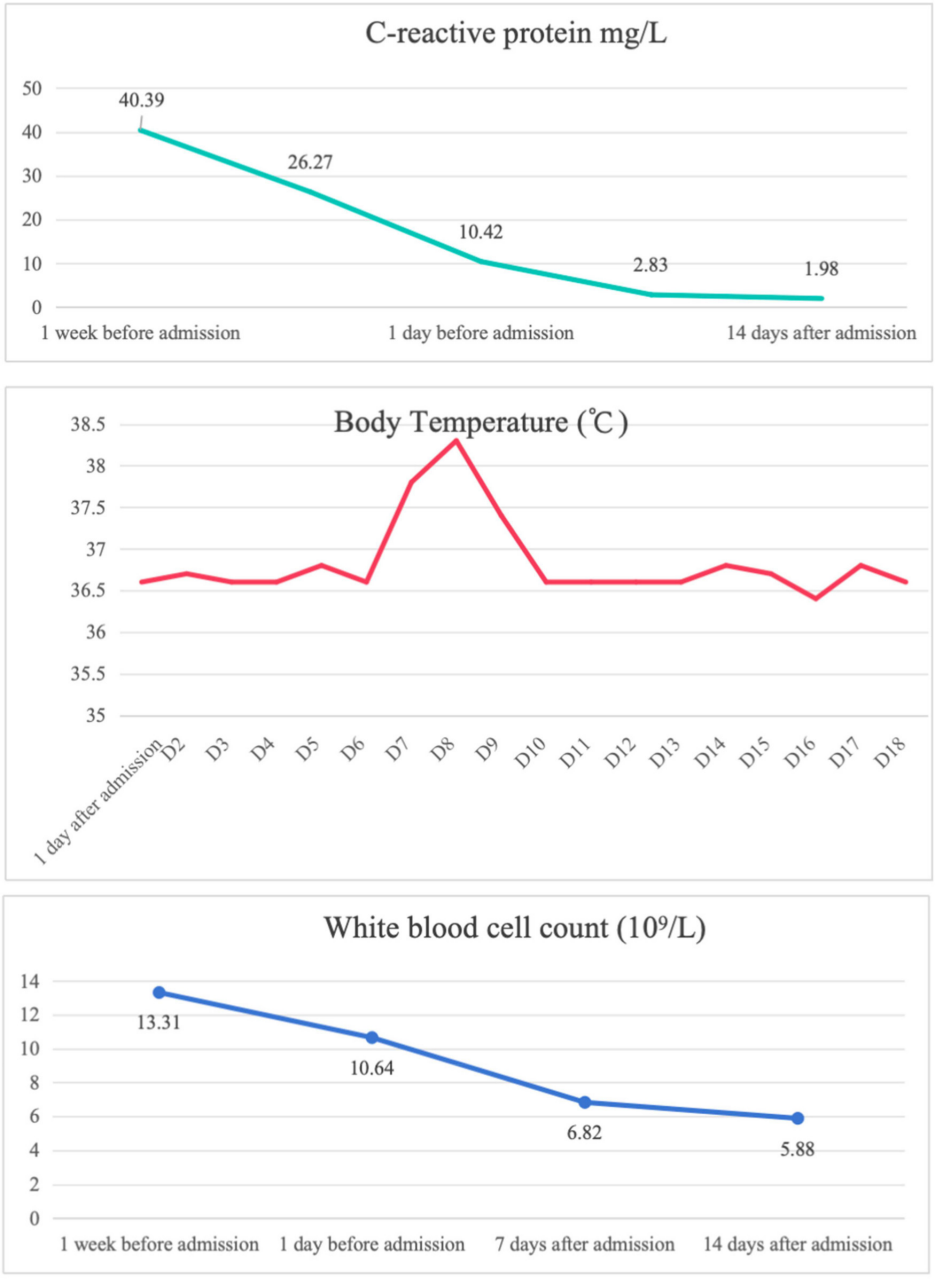
The child's hematological laboratory parameters and serial body temperature profile.

The child was evaluated in our infectious disease clinic on August 7, 2024, where a neck ultrasound revealed bilateral suppurative lymphadenitis (Figure 1A), leading to hospitalization on August 8, 2024. Laboratory workup during admission showed: erythrocyte sedimentation rate (ESR) 39 mm/h and positive nucleic acid detection for *Streptococcus pneumoniae* and *Haemophilus influenzae* in respiratory pathogen testing. Procalcitonin, immunoglobulin levels, PPD testing, and blood cultures were unremarkable or negative. A provisional diagnosis of acute suppurative lymphadenitis was established, and empirical antibiotic therapy with cefoperazone-sulbactam was initiated. Follow-up ultrasound on August 12, 2024, showed no significant improvement in lymphadenopathy (Figure 1B). Repeat laboratory testing on August 15, 2024, demonstrated persistent inflammation (CRP: 26.27 mg/L, ESR: 43 mm/h) with markedly elevated interleukin-6 (44.47 pg/ml) and interleukin-10 (22.15 pg/ml). Due to inadequate clinical response, ultrasound-guided aspiration of the right cervical abscess was performed on August 15, 2024 (Figure 1C). While Gram staining and cultures of the aspirate were negative, mNGS identified 23,255 sequence reads of *L. pneumophila* with 21.07% genome coverage (Table 1 and Figure 2). Antimicrobial therapy was adjusted to intravenous azithromycin and trimethoprim-sulfamethoxazole. Although the neck mass softened and reduced in size, ultrasound-guided re-aspiration was required on August 23, 2024, due to persistent liquefaction. After 18 days of hospitalization, the child was discharged on August 26, 2024, with continued oral azithromycin and trimethoprim-sulfamethoxazole. However, trimethoprim-sulfamethoxazole was discontinued after 8 days due to allergic reaction, and therapy was successfully completed with oral rifampin. The child patient was treated with azithromycin for a total of 14 days, starting from intravenous injection for 7 days and oral administration for 7 days on August 17, 2024. Rifampicin was taken orally for a week, starting from August 30, 2024. During hospitalization, the patient demonstrated progressive improvement in laboratory and clinical parameters, including normalization of white blood cell count, reduction in CRP levels, resolution of fever, and stabilization of other inflammatory markers (Figure 3).

**Table 1:** Results of mNGS in the cervical lymph node pus of the patient.

Type	Genus			Species		
	Name	Relative abundance	Sequence Number	Name	Assess credibility	Sequence number
G-	*Legionella*	99.7%	23,599	*Legionella pneumophila*	99%	23,255

## Discussion

To date, reports of Legionella-induced soft tissue infections remain rare and predominantly involve immunocompromised individuals. We conducted a systematic literature review using PubMed to identify cases of soft tissue infections caused by *Legionella* species. The search identified two notable cases: A 73-year-old woman with nephrotic syndrome and IgA gammopathy of undetermined significance presented with recurrent soft tissue abscesses in the mandible, wrist, and arm. PCR and specialized *Legionella* culture confirmed *Legionella cincinnatiensis* as the pathogen. The patient achieved full recovery after treatment with clarithromycin and rifampin (5). Another one is a 39-year-old woman developed necrotizing soft tissue infection in her left arm caused by *Legionella micdadei* while on immunosuppressive therapy following renal transplantation for polycystic kidney disease (6).

Current first-line antibiotics for Legionella infections include fluoroquinolones, macrolides, and tetracyclines. Additional options such as tigecycline, trimethoprim-sulfamethoxazole, and rifampin may also be utilized in specific clinical scenarios (7). In this case, the combination of a macrolide and rifampin demonstrated favorable therapeutic outcomes.

In this case, we utilized mNGS for the detection of *Legionella*. mNGS is a high-throughput sequencing-based technology that enables comprehensive identification of pathogen-derived nucleic acids directly from clinical specimens. In recent years, driven by reduced sequencing costs and advancements in bioinformatics, mNGS has seen rapid adoption in clinical medicine. Its applications span infectious diseases, oncology, microbiome research, and emerging pathogen discovery, offering unparalleled diagnostic utility for detecting fastidious or unculturable microorganisms (8).

Traditional pathogen detection methods-including culture, antigen/antibody assays, and PCR-face limitations such as prolonged turnaround times, low sensitivity, and narrow diagnostic scope. In contrast, the core strength of mNGS lies in its “hypothesis-free” approach, requiring no prior assumptions about potential pathogens. This makes it particularly valuable for identifying rare or novel pathogens, polymicrobial infections, unculturable organisms, and extrapulmonary infections (9).

In this case, conventional cultures and serological testing failed to identify the causative agent. However, mNGS analysis of lymph node aspirate detected a high sequence read count of Legionella pneumophila, providing critical evidence to guide targeted therapy. The newly developed pathogen capture metagenomic detection technology is based on pathogen metagenomics and utilizes probe hybridization to capture target pathogens (10, 11).

This technology detects nucleic acids in samples and identifies suspected pathogenic microorganisms present in the samples. The detectable range includes 9,945 bacteria with known genome sequences (including 144 mycobacteria and 107 mycoplasma/chlamydia), 6,761 viruses (including DNA and RNA viruses), 1,551 fungi, and 305 parasites. This test includes 54 common pathogenic bacteria, including *Staphylococcus aureus, Streptococcus pneumoniae, Haemophilus influenzae, Pseudomonas aeruginosa, Acinetobacter baumannii*, and so on. Based on the mNGS report, we found that the main pathogenic bacteria of the patient were L. pneumophila.

Beyond infectious diseases, mNGS is gaining prominence in oncology for applications such as tumor-associated pathogen screening, comprehensive tumor genomic profiling, and liquid biopsy-based cancer monitoring (12). Furthermore, mNGS plays an indispensable role in public health emergencies. During the 2019 Wuhan COVID-19 outbreak, Chinese scientists leveraged mNGS to rapidly sequence and publicly share the full SARS-CoV-2 genome within days, laying the foundation for global pandemic response efforts (13).

## Conclusion

This case represents a documented instance of *L. pneumophila* causing suppurative lymphadenitis in a pediatric patient, expanding the known pathogenic spectrum of *Legionella* species. Furthermore, the detection of abundant pathogen nucleic acid through mNGS analysis of purulent fluid overcame the limitations of conventional diagnostic methods, highlighting its critical role in identifying rare or fastidious pathogens.

## Data Availability

The raw data supporting the conclusions of this article will be made available by the authors, without undue reservation.

## References

[1] Gonçalves IG Fernandes HS Melo A Sousa SF Simões LC Simões M. LegionellaDB—a database on Legionella outbreaks. Trends Microbiol. (2021) 29(10):863–6. 10.1016/j.tim.2021.01.015 33612398

[2] Salinas MB Fenoy S Magnet A Vaccaro L Gomes TD Hurtado C Are pathogenic Legionella non-pneumophila a common bacteria in water distribution networks? Water Res. (2021) 196:117013. 10.1016/j.watres.2021.117013 33813251

[3] Phin N Parry-Ford F Harrison T Stagg HR Zhang N Kumar K Epidemiology and clinical management of Legionnaires’ disease. Lancet Infect Dis. (2014) 14(10):1011–21. 10.1016/S1473-3099(14)70713-3 24970283

[4] Gu W Miller S Chiu CY. Clinical metagenomic next-generation sequencing for pathogen detection. Annu Rev Pathol. (2019) 14:319–38. 10.1146/annurev-pathmechdis-012418-012751 30355154 PMC6345613

[5] Gubler JG Schorr M Gaia V Zbinden R Altwegg M. Recurrent soft tissue abscesses caused by Legionella cincinnatiensis. J Clin Microbiol. (2001) 39(12):4568–70. 10.1128/JCM.39.12.4568-4570.2001 11724886 PMC88590

[6] Kilborn JA Manz LA O'Brien M Douglass MC Horst HM Kupin W Necrotizing cellulitis caused by Legionella micdadei. Am J Med. (1992) 92(1):104–6. 10.1016/0002-9343(92)90024-6 1731498

[7] Jasper AS Musuuza JS Tischendorf JS Stevens VW Gamage SD Osman F Are fluoroquinolones or macrolides better for treating Legionella pneumonia? A systematic review and meta-analysis. Clin Infect Dis. (2021) 72(11):1979–89. 10.1093/cid/ciaa441 32296816 PMC8315122

[8] Diao Z Han D Zhang R Li J. Metagenomics next-generation sequencing tests take the stage in the diagnosis of lower respiratory tract infections. J Adv Res. (2021) 38:201–12. 10.1016/j.jare.2021.09.012 35572406 PMC9091713

[9] Zhu Y Gan M Ge M Dong X Yan G Zhou Q Diagnostic performance and clinical impact of metagenomic next-generation sequencing for pediatric infectious diseases. J Clin Microbiol. (2023) 61(6):e0011523. 10.1128/jcm.00115-23 37260394 PMC10281092

[10] Briese T Kapoor A Mishra N Jain K Kumar A Jabado OJ Virome capture sequencing enables sensitive viral diagnosis and comprehensive virome analysis. mBio. (2015) 6(5):e01491–15. 10.1128/mBio.01491-15 26396248 PMC4611031

[11] Metsky HC Siddle KJ Gladden-Young A Qu J Yang DK Brehio P Capturing sequence diversity in metagenomes with comprehensive and scalable probe design. Nat Biotechnol. (2019) 37(2):160–8. 10.1038/s41587-018-0006-x 30718881 PMC6587591

[12] Guo Y Li H Chen H Li Z Ding W Wang J Metagenomic next-generation sequencing to identify pathogens and cancer in lung biopsy tissue. EBioMedicine. (2021) 73:103639. 10.1016/j.ebiom.2021.103639 34700283 PMC8554462

[13] Chen L Liu W Zhang Q Xu K Ye G Wu W RNA Based mNGS approach identifies a novel human coronavirus from two individual pneumonia cases in 2019 Wuhan outbreak. Emerg Microbes Infect. (2020) 9(1):313–9. 10.1080/22221751.2020.1725399 32020836 PMC7033720

